# Image cytometric nuclear texture features in inoperable head and neck cancer: a pilot study

**DOI:** 10.2478/v10019-011-0002-y

**Published:** 2011-02-23

**Authors:** Margareta Strojan-Flezar, Jaka Lavrencak, Mario Zganec, Primoz Strojan

**Affiliations:** 1 Institute of Pathology, Faculty of Medicine, University of Ljubljana, Ljubljana, Slovenia; 2 Department of Cytopathology, Institute of Oncology Ljubljana, Ljubljana, Slovenia; 3 Alpineon d.o.o., Ljubljana, Slovenia; 4 Department of Radiation Oncology, Institute of Oncology Ljubljana, Ljubljana, Slovenia

**Keywords:** head and neck cancer, image cytometry, nuclear features, prognosis

## Abstract

**Background:**

Image cytometry can measure numerous nuclear features which could be considered a surrogate end-point marker of molecular genetic changes in a nucleus. The aim of the study was to analyze image cytometric nuclear features in paired samples of primary tumor and neck metastasis in patients with inoperable carcinoma of the head and neck.

**Materials and methods.:**

Image cytometric analysis of cell suspensions prepared from primary tumor tissue and fine needle aspiration biopsy cell samples of neck metastases from 21 patients treated with concomitant radiochemotherapy was performed. Nuclear features were correlated with clinical characteristics and response to therapy.

**Results:**

Manifestation of distant metastases and new primaries was associated (p<0.05) with several chromatin characteristics from primary tumor cells, whereas the origin of index cancer and disease response in the neck was related to those in the cells from metastases. Many nuclear features of primary tumors and metastases correlated with the TNM stage.

**Conclusions:**

A specific pattern of correlation between well-established prognostic indicators and nuclear features of samples from primary tumors and those from neck metastases was observed. Image cytometric nuclear features represent a promising candidate marker for recognition of biologically different tumor subgroups.

## Introduction

Various tumor variables related to DNA content were found predictive for the response of different tumor types to applied therapy; they also contain prognostic information on patients’ survival.^1^–^3^ Image or flow cytometry can be used as a fast and effective method for the measurements of DNA content and S-phase to evaluate the occurrence and to quantify genetic changes in different malignancies. In addition, image cytometry can measure numerous nuclear features and the foremost nuclear texture features on a two-dimensional snapshot of chromatin structure and organization. Accordingly, nuclear texture features could be considered a surrogate end-point marker of molecular genetic changes in a nucleus.^4^ In the head and neck region, image cytometry has principally been used to detect and assess premalignant changes.^5^–^8^ However, a recent study reported that image cytometric DNA content and nuclear morphometric features were significantly associated with the radiosensitivity of nasopharyngeal carcinoma and outcome of the disease.^9^

In the present study we aimed to analyze image cytometric nuclear features (foremost nuclear texture features) in paired tissue samples of primary tumors and regional metastases from the neck in a group of patients with inoperable squamous cell carcinoma of the head and neck (SCCHN) treated with concomitant chemoradiotherapy with mytomycin C and cisplatin. We hypothesized that chromatin characteristics as determined by image cytometry could improve assessment of the biological potential of SCCHN reflected in the response to treatment and course of the disease.

## Materials and methods

### Patients

The study group comprised 21 patients with inoperable SCCHN who entered the phase I/II clinical study between 2002 and 2004.^10^ Their median age was 57 years (range, 38–68). TNM stage was IVA in 6 patients and IVB in 15 patients. Details on patients and their tumors are presented in Table 1. All patients were treated with curative intent using a uniform protocol of concomitant chemoradiotherapy consisting of conventional radiotherapy (70 Gy in 35 fractions) and concomitant Mitomycin C, bioreductive agent, selectively toxic for hypoxic cells, applied I.V. in a dose of 15 mg/m^2^ after the delivery of 10 Gy. Additionally, Cisplatin at a dose of 10 mg/m^2^/day (6 patients) or 14 mg/m^2^/day (15 patients) I.V., was applied during the last 10 fractions of irradiation to counteract the accelerated repopulation of surviving tumor clonogens (“chemoboost”). The details on treatment protocol were published elsewhere.^10^

The study protocol was approved by the National Medical Ethics Committee of the Republic of Slovenia, and informed consent was obtained from all patients participating in the study.

### Preparation of tissue samples

Fifty-micron sections were cut from the formalin-fixed paraffin-embedded tissue blocks of primary tumors biopsied during diagnostic assessment. Single-cell suspensions were prepared according to the standard procedures.^11^,^12^ For image cytometric analysis, the cell suspension was filtered through a membrane filter system and the filter was gently imprinted onto two parallel glass slides. Cell preparations were immediately immersed in Delaunay fixative (ethanol:acetone 1:1 with 0.5 ml/l trichloracetic acid).

Cell samples of neck metastases were obtained during diagnostic procedures before treatment by fine needle aspiration biopsy (FNAB) using a 20-gauge (0.7 mm diameter) needle. A direct smear was prepared from each aspirate, air-dried and stained according to Giemsa for diagnostic light microscopy evaluation. The needle and syringe were then washed in cell medium (containing 4.5% bovine serum albumin, 0.45% EDTA in PBS with 1 IU/1ml penicillin and stored at 4°C). This resulted in cell suspension that was used to prepare several cytospins in a Shandon cytospin 4 cytocentrifuge (Thermo Shandon Inc, Pittsburgh, Pennsylvania, U.S.A.). Cytospins were fixed in Delauney fixative. For image cytometric measurements, the slides prepared from cell suspensions of primary tumors and metastases were stained stoichiometrically with thionin according to the Feulgen method.^13^

### Image cytometric analysis

Image cytometric analysis was performed using an automated high resolution image cytometer, Cyto-Savant™ (Oncometrics Imaging Corp., Vancouver, BC, Canada).^14^ The slides were automatically scanned with the software program Acquire (Oncometrics Imaging Corp., Vancouver, BC, Canada), incorporated into the system. More than 100 nuclear features were calculated from each of the nuclear images (Table 2). The nuclear features included common morphometric features (area, diameter, shape features, etc.), descriptive statistics of chromatin distribution (integrated optical density [IOD], variance of optical density [OD], OD skewness and kurtosis, etc.) and nuclear texture features, represented by discrete chromatin distribution (area and shape of high, medium and low density chromatin components, average distance between chromatin components of the same optical densities, etc.), fractal texture features, Markovian texture features (entropy, energy, correlation, homogeneity, cluster shade, etc.), non-Markovian local extreme features (number of local minima and maxima in the image, etc.) and the length of texture features’ run (short run emphasis, long run emphasis, gray level nonuniformity, etc.). The formulas and nuclear feature descriptions can be found elsewhere.^15^

### Statistics

Probability density functions were calculated for each nuclear feature in each tumor and metastasis, respectively.^16^ Differences between the study groups were evaluated with analysis of variance. Differences were considered statistically significant at p<0.05.

## Results

### Patients, image analysis, therapy and outcome

In 21 patients, after interactive removal of artifacts and double or multiple nuclei, an average of 2851 well-preserved nuclear images per slide (range, 91–8158) were available for image cytometric analysis of tissue samples of primary tumors, and an average of 1880 nuclear images per slide (range, 50–8563) in the FNAB samples of neck metastases.

The median follow-up time was 49 months (range, 38–63). At 8–12 weeks post therapy, complete response to treatment at the primary site and in the neck disease was observed in 19 (90.5%) and 16 (76.2%) patients, respectively. The disease reappeared locally in 2/19 patients and regionally in 5/16 patients who obtained a complete response at the primary site and in the neck, respectively. Six (28.6%) patients were diagnosed with distant metastases, and new primary tumors developed in six (28.6%) patients, 3–46 months (median, 18 months) post diagnosis of index head and neck carcinoma.

### Nuclear features

Differences in nuclear features in relation to various clinical parameters and treatment outcome are summarized in Table 3, showing a specific pattern of correlation between individual prognostic indicators and nuclear features of samples from primary tumors and those from neck metastases.

Direct comparison of nuclear texture features between the primary tumors and metastases was not prudent due to initial differences in primary fixation procedures of the two sample types.^17^

### Primary tumor

In regard to the primary tumor site, image cytometric analysis of nuclear features showed no differences when primary tumor samples were analyzed. Considering the TNM stage of the disease, differences were found in some of the nuclear features, namely photometric, Markovian and non-Markovian. Only one of the studied nuclear texture features (non-Markovian) was predictive of regional recurrence by analyzing the primary tumor but none of them forecasting nodal response to therapy. The nuclear features of primary tumors belonging to all groups of nuclear morphometric and texture features differed between the patients who developed distant metastases and those who didn’t. Also, some nuclear texture features of primary tumors were different for the patients who developed new primary tumors.

### Neck metastasis

**FIGURE 1.**
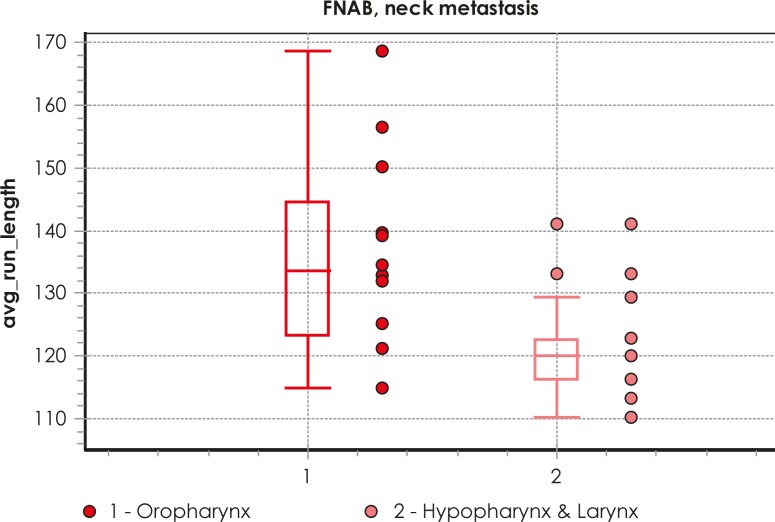
Example of box plot of different values of one nuclear texture feature (avg_run_length) for primary tumor site as analyzed in FNAB samples from neck metastases.

Differences between several nuclear morphometric and texture features (discrete, non-Markovian and run length) were found in the samples from metastases when patients were grouped according to the primary tumor site (Figure 1). When analyzing samples from patients with disease TNM stage IVA and stage IVB, the differences were also found for some nuclear texture features (discrete, non-Markovian). Variations in regional response to treatment at 8–12 weeks post therapy were associated with some morphometric and other nuclear features (photometric, discrete, fractal and run length) of metastases. Only one nuclear photometric feature was predictive for systemic dissemination of the disease, while no differences were observed in any feature of metastases in regard to regional recurrence or occurrence of new primary tumor.

## Discussion

In the present study we aimed to evaluate the predictive and prognostic value of image cytometric nuclear features, characterizing different details of nuclear size, chromatin structure and its organization, in matched pairs of primary SCCHN and neck metastasis in a homogenous group of patients, in regard to the tumor extent and therapy.^10^ The main finding would be that nuclear features as determined in primary tumor samples suggest the tendency for distant dissemination and occurrence of new primary tumors. On the other hand, the nuclear features of neck metastases determined regional response of the neck disease to applied radiochemotherapy and indicated primary tumor origin.

To the best of our knowledge, our study is the first to analyze the clinical applicability of image cytometric assessment of nuclear features, including texture features, in advanced SCCHN. Other research on head and neck lesions has focused on the difference between malignant and non-malignant tissue characteristics, aiming to recognize a pattern predictive of the malignant transformation of clinically normal appearing mucosa or premalignant changes.^5^–^7^ The results of these studies suggested that nuclear features could discriminate between benign and malignant mucosal changes, but further studies on their potential to predict the probability of malignant transformation of benign lesions were not conducted. Furthermore, malignancy-associated changes recognized by image cytometric analysis of non-malignant buccal mucosa cells could be used to identify high-risk individuals for invasive cancer, including SCCHN.^18^,^19^

Our study revealed various associations with clinically well-established prognostic factors. It is of interest that an individual prognosticator correlated with the nuclear features of a particular sample type, *i.e.* either primary tumor biopsy samples or regional metastasis FNAB samples. Although sharing many characteristics, various factors from local environment, which differed between primary tumor site and the neck, most probably critically and distinctly influence tumor cells forming primary tumor and nodal metastasis.^20^ Thus, it sounds reasonable that FNAB cell samples obtained directly from metastatic nodes are more representative than biopsy specimens from primary tumors for characterizing nodal disease and vice versa. The lack of effect of T-stage on the risk of nodal failure^21^ and, conversely, the observation that nodal disease at presentation does not add any significant contribution to the risk of local failure^22^ corroborate this assumption.

We observed that manifestation of distant metastases and new primary tumors was significantly dependent on several nuclear chromatin features from primary tumor cells. It seems that the biological potential of cells from primary tumors characterize the clinical aggressiveness of the disease and dictate its course in an individual patient. On the other hand, the origin of index cancer and disease response to treatment in the neck at 8–12 weeks post therapy were related to nuclear features as characterized in the cells from neck metastases. Obviously, the nuclear chromatin characteristics of tumor cells from regional metastases reflect their origin, as well as susceptibility to chemoradiation. In our previous study we found FNAB sampling of neck metastases of SCCHN supplemented with immunocytochemical determination of cyclin D1 and Ki-67 a valuable method for radiosensitivity testing to predict regional response to radiotherapy.^23^ Likewise, Cabanillas *et al*. reported on a significant correlation between p53 expression in the primary SCCs of the supraglottic larynx and the matched lymph node metastases, although only p53 over-expression in the lymph nodes was predictive for regional recurrence.^24^ To the contrary, Huang *et al*. found nuclear morphometry of primary tumor cells in patients with nasopharyngeal carcinoma assessed by image cytometric analysis to be predictive for the response of the primary tumor and regional metastases to radiotherapy.^9^

None of the nuclear features tested correlated with the progression of regional disease in our patients; the putative explanation would be that in the original samples taken before therapy, small clones of resistant cells surviving the treatment were not sampled and, consequently, not analyzed. This assumption goes into the context of the extremely advanced disease stages in our patients (T4 – 71.4%, N3 – 57.1%; all patients had stage IV disease), which increases the probability of under-representative samples (*i.e.* with an insufficient number of representative cells). Furthermore, disease curability could be also influenced by tumor burden and radiotherapy related factors *i.e*. technique and dose. On the other hand, many nuclear features of both primary tumors and metastases correlated with the stage of the disease, reflecting the fact that both contribute to the TNM-stage grouping.

One important advantage of image cytometric analysis is that data on nuclear features can be obtained before treatment, so they could be used for its planning. Because in SCCHN there are no markers identified so far to reliably predict the course of the disease, its response to therapy or patients’ survival, our findings suggest image cytometric evaluation of the primary tumor and/or its metastases as a plausible prognostic tool. Showing the potential to be able to discriminate between different forms of the disease in respect to its aggressiveness, the nuclear features assessed by image cytometry emerge as a valuable method for tailoring treatment accordingly.

When evaluating the clinical relevance of the presented results, one must be aware of existing drawbacks of the present study. First, the number of patients is small, which precluded more detailed statistics. Furthermore, although being homogenous regarding disease stage and therapy, the patients differed significantly in respect to primary tumor origin, which could be of prognostic significance.^25^ And finally, using pairs of cell samples obtained from primary tumors and their regional metastases, we gained a unique opportunity to compare chromatin characteristics between the cells of index cancers and their metastases. Unfortunately, due to the differences in primary fixation procedures, which influence the nuclear features of the two sample types, as we already proven previously, this was inappropriate in the present study.^17^

In conclusion, a specific pattern of correlation between well-established prognostic indicators and nuclear features of samples from primary tumors and those from neck metastases was observed. According to our results, image cytometric nuclear features represent a promising candidate marker for the recognition of biologically more aggressive tumors and could add to a more individualized treatment of SCCHN patients. However, the study should be considered as the hypothesis generated; it was designed as a pilot series, asking for, on the basis of favorable results, confirmation in a large-scale prospective clinical trial.

## Figures and Tables

**TABLE 1.** Characteristics of patients and their tumors

**Patients**						
Gender			female – 1, male – 20			
Age			57 (38–68)^a^			
Primary tumor site						
Oropharynx			12			
Hypopharynx			7			
Larynx			2			
Histopatological grade						
Grade 2			15			
Grade 3			6			
UICC TNM classification						
	N1	N2A	N2B	N2C	N3	Total

T1					1	1
T2					3	3
T3					2	2
T4A			3	2	5	11
T4B	1		1	2	1	4

Total	1	0	4	4	12	21
Overall UICC-TNM stage						
Stage IVA			6			
Stage IVB			15			

a Median (range), in years

**TABLE 2.** Image cytometric nuclear features

**Nuclear Feature Group**	**Specific Nuclear Feature**
Morphometric nuclear features	area, mean radius, variance of radius, sphericity, eccentricity, elongation
Photometric Nuclear Features (descriptvive statistics of chromatin distribution)	DNA Amount, DNA Index, variance of DNA intensity, mean DNA intensity, OD mean, OD maximum, OD variance, OD scewness, OD curtosis
Discrete Nuclear Texture Features	low density DNA area, medium density DNA area, high density DNA area, low density DNA amount, medium density DNA amount, high density DNA amount, low density DNA compactness, medium density DNA compactness, high density DNA compactness, medium/high density DNA compactness, low density - average distance, medium density - average distance, medium/high density average distance, low density objects, medium density objects
Markovian Nuclear Texture Features	entropy, energy, contrast, correlation, homogeniety, cluster shade, cluster prominence
Non-Markovian Nuclear Texture Features (local extreme features)	density light spot, density dark spot, range average, range extreme, center of gravity
Fractal Nuclear Texture Features	fractal1 area, fractal2 area, fractal dimension
Run Length Nuclear Texture Features	average long runs, average run length, short run emphasis, long run emphasis, grey level nonuniformity

**TABLE 3.** Differences in nuclear features determined in tissue samples from primary tumors and neck metastases, according to clinical characteristics and treatment outcome. Differences at p<0.05 are marked with +

**Nuclear features**	**Clinical characteristics of patients**											
	**Primary tumor site**		**TNM stage**		**Regional response^1^**		**Regional recurrence**		**Distant metastases**		**New primary tumor**	
	**PT**	**NM**	**PT**	**NM**	**PT**	**NM**	**PT**	**NM**	**PT**	**NM**	**PT**	**NM**
Morphometric		+				+			+		+	
Photometric			+			+			+	+		
Discrete		+		+		+			+		+	
Markovian			+						+			
Non-Markovian		+	+	+			+		+		+	
Fractal						+			+		+	
Run-length		+				+						

PT, Primary tumor; NM, Neck metastasis

^1^ At 8–12 weeks post-therapy
